# Estimating the Duration of Pertussis Immunity Using Epidemiological Signatures

**DOI:** 10.1371/journal.ppat.1000647

**Published:** 2009-10-30

**Authors:** Helen J. Wearing, Pejman Rohani

**Affiliations:** 1 Department of Biology and Department of Mathematics & Statistics, University of New Mexico, Albuquerque, New Mexico, United States of America; 2 Odum School of Ecology and Center for Tropical and Emerging Global Diseases, University of Georgia, Athens, Georgia, United States of America; 3 Fogarty International Center, National Institutes of Health, Bethesda, Maryland, United States of America; Emory University, United States of America

## Abstract

Case notifications of pertussis have shown an increase in a number of countries with high rates of routine pediatric immunization. This has led to significant public health concerns over a possible pertussis re-emergence. A leading proposed explanation for the observed increase in incidence is the loss of immunity to pertussis, which is known to occur after both natural infection and vaccination. Little is known, however, about the typical duration of immunity and its epidemiological implications. Here, we analyze a simple mathematical model, exploring specifically the inter-epidemic period and fade-out frequency. These predictions are then contrasted with detailed incidence data for England and Wales. We find model output to be most sensitive to assumptions concerning naturally acquired immunity, which allows us to estimate the average duration of immunity. Our results support a period of natural immunity that is, on average, long-lasting (at least 30 years) but inherently variable.

## Author Summary

The eradication of vaccine-preventable infectious diseases remains an important public health priority. To achieve this goal, the level of immunity afforded needs to be high and long-lasting. For pertussis, one of the leading causes of mortality in infants, immunity has been shown to wane in some individuals. The epidemiological impacts of this observation depend critically on the duration of protective immunity in the entire population, which remains notoriously difficult to estimate. We approach this problem by exploring the agreement between model dynamics and case notification data from England & Wales. Our estimates suggest the average duration of immunity is much longer than is currently thought (at least 30 years), but that some individuals would lose immunity quite rapidly.

## Introduction

Pertussis has been an important cause of morbidity and mortality for centuries [1] and remains a significant cause of infant mortality worldwide [2]. During the 1940s and 1950s, many industrialized countries implemented widespread pertussis immunization programmes, which resulted in dramatic declines in disease incidence. In the last decade, however, a growing number of highly vaccinated countries, such as the US [3], Canada [4], France [5] and the Netherlands [6], have reported an increasing trend in the general incidence of pertussis. This potential resurgence has raised serious concerns about the effectiveness of current pertussis vaccination strategies [7]–[9] and whether pertussis eradication is an achievable goal [10]. Our understanding of pertussis epidemiology has been complicated by the accumulation of evidence that in some individuals the immunity acquired from natural infection is not permanent [11]–[13], as was traditionally postulated [14],[15]. The upsurge in reported incidence has led to the hypotheses that loss of immunity to pertussis is more widespread than previously thought, that vaccine-induced immunity may wane more rapidly than that acquired from natural infection, and that vaccination may have a greater impact on the severity of disease rather than on the transmission of infection.

Accurate assessment of the duration of immunity after natural infection or vaccination is crucial for pertussis control, and yet our understanding of immunity to pertussis is limited. The central obstacle is that despite a great deal of clinical research, it remains impossible to correlate protection against pertussis with a quantifiable immune response against a single protective antigen [16]–[18]. This is partly because, in contrast to other vaccine-preventable bacterial infections, such as diphtheria or tetanus, where antibodies are known to protect against the toxin that mediates disease, pertussis produces a range of toxins including pertussis toxin, endotoxin, adenylate cyclase toxin and tracheal cytotoxin, which are known to play a role in pathogenesis and immune evasion [19]. Immunity to pertussis is further complicated by the production of numerous virulence factors (filamentous hemagglutinin, pertactin and fimbriae) that aid bacterial persistence in the respiratory tract. Moreover, in addition to binding to epithelial cells in the respiratory tract (which facilitates extracellular multiplication), pertussis also survives within macrophages and other cell types, an observation that argues for a role for cell-mediated as well as humoral immunity in protection [19],[20].

A recent review by Wendelboe *et al.* [21] of the handful of published studies on duration of immunity suggested estimates in the range 7–20 years for naturally acquired immunity and 4–12 years for vaccine-induced immunity against disease. The wide range in estimates may be due to a combination of differences in study methodology and pertussis epidemiology in different countries. Recent estimates of naturally acquired immunity are generally based on a very small set of studies conducted in the vaccine era. Estimates of vaccine-induced immunity are often difficult to make because vaccine efficacy (primary vaccine failure) and waning immunity (secondary vaccine failure) are confounded, and potentially affected by variation in vaccine content, manufacture and schedule.

Given the challenges in understanding pertussis immunity using clinical approaches, and the limitations of using cohort and case series studies, a number of researchers have studied pertussis transmission models to explore how waning immunity influences pertussis epidemiology in the vaccine era [22],[23], paying particular attention to its consequences for the age-specific serological profile [24] and the severity of disease [25]. However, a systematic assessment of the degree of waning immunity that is consistent with temporal and spatial incidence data, both in the pre-vaccine and vaccine era, is lacking. As a first step toward achieving this, we need testable predictions about the various dynamical signatures we may expect to observe in epidemiological data, such as the temporal patterns of outbreak dynamics, spatial synchrony and fade-out structure. Here, we outline a simple model for pertussis immunity and transmission and compare predictions with epidemiological data for two key dynamical metrics–inter-epidemic period and critical community size, defined as the minimum population size above which pertussis remains endemic.

## Methods

### A model for pertussis immunity and transmission

**Figure 1:**
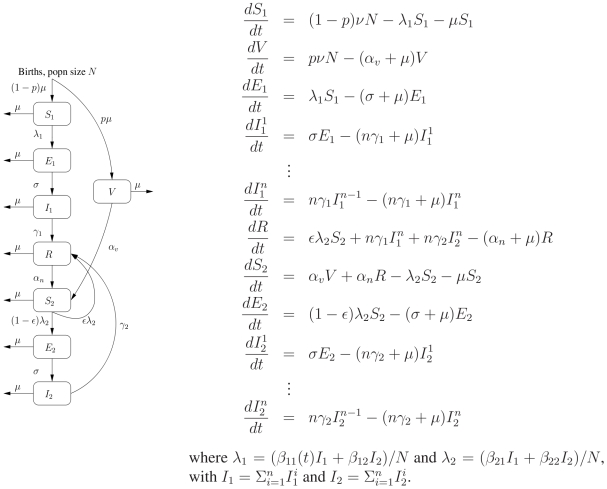
A model of pertussis immunity and transmission: an extension of the SEIR paradigm that allows for reinfection and gamma-distributed infectious periods. The parameter 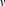 denotes the background birth rate, 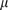 the background death rate, 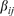 is the transmission rate from infected individuals *I_j_* to susceptible individuals *S_i_*, and 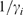 is the average length of infectiousness, where *i*, *j* = 1 represents primary infections and *i*, *j* = 2 represents repeat infections. To mimic the opening and closing of schools, which affects transmission between children [40], we assume that 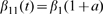 during term time and 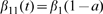 during school holidays [41]. In addition, because we would like to focus on the relative infectiousness of repeat infections to primary infections (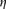), and the relative magnitude of the contact rates, we rewrite the transmission rates as 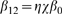, 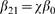 and 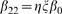, where 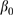 is the average transmission rate from individuals with a primary infection to naive individuals. Following the work of Nguyen & Rohani [42], we assume that the infectious period is gamma-distributed with shape parameter *n* = 4. The parameter 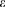 represents the probability that susceptible (but previously infected or vaccinated) individuals, upon exposure, boost their immunity instead of becoming infectious. In the basic model, 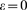 and in the immune-boosting model, 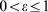.

To study the importance of waning immunity in shaping the epidemiology of pertussis, we adopt an extension of the classic Susceptible-Exposed-Infectious-Recovered (SEIR) paradigm that accounts for reinfection and the possibility of both primary and repeat infections. In Figure 1 we illustrate how the compartmental model is set up, along with the system of deterministic differential equations that formally describe the dynamics. We divide the susceptible population into those who are naive to exposure (*S*_1_) and those who have previously been infected or vaccinated (*S*_2_). Similarly, exposed and infected individuals are divided into those who are experiencing primary (*E*_1_, *I*_1_) and repeat infections (*E*_2_, *I*_2_) [24], while the variable, *R*, represents all those who are recovered and temporarily protected from natural infection. To incorporate vaccination, we assume that a proportion, *p*, of newborns is successfully immunized and enters the vaccinated class (*V*). The parameter *p*, therefore, is a composite measure taking into account both vaccine uptake and primary vaccine failure. Immunized individuals may lose their immunity and become susceptible (*S*_2_). We examine two distinct scenarios relating to the fate of *S*_2_ individuals who become exposed. First, in our basic model, we assume they may experience repeat infections in a manner similar to those who acquire immunity from natural infection. Or, second, in our immune-boosting model, we assume that with probability 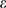, exposure may boost their immunity whereupon they revert to the temporary immune class, *R*.

Distinguishing between primary and repeat infections will allow us to consider how variations in disease severity (*e.g.* if repeat infections are less symptomatic than primary infections) could potentially affect reported case numbers. The distinction between infection and disease plays a fundamental role in the hypothesis that natural or vaccine-induced immunity is not permanent. Most severe cases of typical pertussis still occur in the very young (infants who are not yet immunized), which suggests that if repeat bouts of infection occur, they result in reduced disease [25]. Because most reported cases are those exhibiting clinically presenting symptoms, a key issue is how important unknown infections are for pertussis transmission and thus persistence. There are potentially two unseen cohorts: those with atypical symptoms such as a persistent cough, who are probably contributing to transmission; and those with asymptomatic infections, who are less likely to be contributing to transmission but are boosting their own immunity and important to herd immunity. In the model, loss of immunity is incorporated in a very simple manner: we assume that immunity, although temporary, is complete, and that it is lost at a constant rate represented by 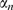 for naturally acquired immunity and 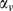 for vaccine-induced immunity.

To explore the consequences of different assumptions about waning immunity and the role of repeat infections, we develop a stochastic event-driven analogue of the model presented in Figure 1 (see, for example, [26]). Specifically, we transform our deterministic model into its stochastic analogue using an approximation to Gillespie's direct algorithm [27] known as the τ-leap method [28],[29]. We use a time-step of 0.001 year (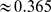 day), which was found to yield significant speed-up without sacrificing accuracy when compared to Gillespie's direct algorithm for some initial test cases. We simulate the model for various population sizes as key immunity and transmission parameters are systematically varied. This allows us to investigate the realized inter-epidemic period and critical community size in both the pre-vaccine and vaccine eras.

### Parameter values

An important initial step in generating model predictions is determining parameter values. Certain epidemiological and demographic characteristics, such as the average length of the latent and infectious periods [30] and the average life expectancy (death rate^−1^) are relatively well defined by independent data (these parameter values are fixed throughout our investigations and their values are listed in Table 1). In addition, we calculate estimates of the range of birth rates over the period from demographic data on the England and Wales cities. For simulation purposes, we randomly choose a rate from the range in the pre-vaccine era (and then decrease this in the vaccine era) for each realization. With a fixed death rate, this leads to a non-stationary population size that may affect the force of infection through frequency-dependent transmission. To understand how this might change model predictions, we conducted simulations in two different ways: one where we recalculated the population size to use in the force of infection, and another where we kept it fixed at the initial size. For the range of birth rates used, and the length of time series analyzed, both methods yielded very similar results.

**Table 1:** Description and baseline values of parameters for the model. These are the values used unless otherwise stated.

Parameter	Epidemiological description	Value
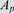 (pre-vaccine era)	Average age at first infection	4 years
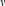 (pre-vaccine era)	Birth rate	(0.012,0.028) year^−1^
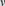 (vaccine era)	Birth rate	pre-vaccine rate - 0.002
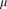	Death rate	1/70 year^−1^
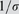	Average latent period	8 days
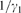	Average infectious period for primary infections	15 days
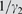	Average infectious period for repeat infections	15 days
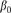	Average transmission rate from 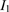 to 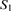	see Appendix S1
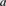	Relative amplitude of seasonal forcing	0.15
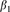	Parameter in forcing function constrained	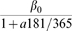
	so that the average transmission rate is 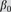	
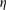	Relative infectiousness of repeat	1
	to primary infections	
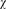	Ratio of contact rate between children and	0.5
	adults to that between children	
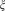	Ratio of contact rate between adults	0.75
	to that between children	
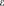	Immune-boosting probability	0 or 0.5
*p* (pre-vaccine era)	Proportion of newborns successfully vaccinated	0
*p* (vaccine era)	Proportion of newborns successfully vaccinated	0.6

By contrast, direct estimation of transmission rates is substantially more problematic. In the past, employing results derived from mathematical models, estimates of the average age at (first) infection (*A_p_*) have been used to infer transmission rates [31]. For pertussis, this has resulted in the widely-used estimate of the basic reproductive ratio, 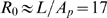 in the pre-vaccine era in England and Wales (based on 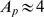 and average life expectancy, 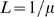, of 70 years). This estimate relies on the assumption of permanent immunity, and so for our model we recalculate transmission rates and *R*_0_ for different assumptions about waning immunity, using the same estimates of *A_p_* and *L* (see Appendix S1 for details). In general, if we fix the average age at infection, waning immunity results in a reduction in the estimated value of *R*_0_, via a reduction in transmission rates. This is entirely intuitive: an infectious disease which does not confer long-lasting immunity does not need to be as transmissible to attain the same prevalence as one that results in permanent immunity, because lower transmission is offset by faster replenishment of the susceptible pool.

A potential pitfall of such an analysis would be to allow parameters to vary independently because changes in assumed immunity characteristics affect the basic reproductive ratio, *R*_0_ (see Appendix S1 for details; [29]). Therefore, in order to ensure our model dynamics conform to important epidemiological observations [31], we constrain our transmission traits as immunity parameters are varied to keep the mean age at primary infection fixed at 4 years.

To compare our model predictions to data, we record new cases (new arrivals to the recovered class) and assume that 15% of primary infections are reported and only 1% of repeat infections (below, we discuss the effects of varying the reporting rate). Our primary reporting rate was calibrated (in the pre-vaccine era and in the absence of reporting repeat infections) so that the number of case reports predicted by the model quantitatively agrees with observed values in the England and Wales data and is supported by the work of Clarkson & Fine [32]. This calibration holds as we vary certain transmission and immunity parameters, because by fixing the age at primary infection, the equilibrium level of primary infections does not depend on the number of repeat infections (see Appendix S1).

## Results

### Inter-epidemic periods

**Figure 2:**
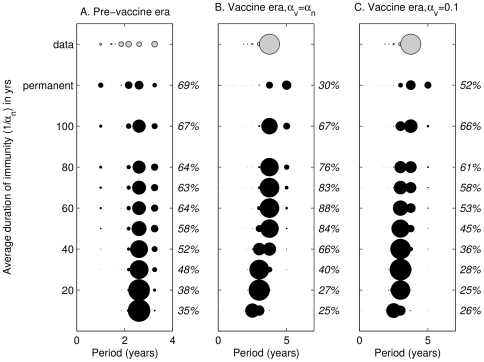
Basic model: analyses of the dominant periods of the England and Wales pertussis data (gray markers) compared to the dominant periods of stochastic realizations of the pertussis reinfection model (black markers), as the duration of immunity is varied. Panel A illustrates results for the pre-vaccine era, panel B for the vaccine era assuming that 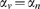, and panel C for the vaccine era fixing the average duration of vaccine-induced immunity at 10 years (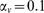). The diameter of the marker reflects the proportion of the 50 largest cities (data) or 1000 simulations (model: 50 realizations are generated for 20 different population sizes) for which spectral analysis of weekly case reports reveals that period to be the dominant signal (note: as we show in Figure S11, increasing the number of stochastic realizations does not qualitatively affect our findings). Any dominant period not significant at the 95% level is denoted as having a period of 0 years. The average normalized power for each dominant signal is illustrated in Figure S1. The length of time series analyzed is 13 years in the pre-vaccine era, and 15 years in the vaccine era. The percentages displayed to the right of each panel are the overlap between the data and the model output. Parameter values for the model are given in Table 1, with 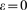. The population size, *N*, is varied from 75,000 to 1.5 million. To allow for the reintroduction of infection following extinction in a single population, we include a background force of infection of 50/million/yr (results are similar if we assume 10/million/yr). The axis representing the average duration of immunity is not to scale between 100 years (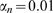) and permanent immunity (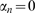).

We begin by comparing model predictions about the inter-epidemic periods to observations from England and Wales in the pre-vaccine era (1945–1957, inclusive) and vaccine era (1958–1972, inclusive) when inter-epidemic periods were in the range 2–3 years and 3–4 years respectively [26],[33],[34]. Figure 2 summarizes the dominant periods of fifty stochastic realizations of the basic model for 20 different population sizes (from 75,000 to 1.5 million, a range which represents all sizes of city in the data) as we vary the duration of immunity in the pre-vaccine and vaccine era. Model results (black markers) are displayed below the same analyses performed on the data (gray markers). The diameter of a marker centered at a particular value of cycle period reflects the proportion of time series for which that period was the dominant signal. For example, in Figure 2A, if we consider the horizontal line of markers corresponding to an average duration of immunity of 10 years, we can see that most of the realizations result in time series with a detectable dominant period of around 2.5 years. For each set of realizations, represented by a different duration of immunity, we compare the set of dominant periods to those found in the data. The percentage overlap between the two sets is then displayed to the right in each panel.

The observed and predicted periods exhibit variation due to variability in birth rates, population sizes and stochastic effects. In the pre-vaccine era, variation in the predicted inter-epidemic period and the influence of annual term-time forcing decreases as the duration of immunity is reduced. The parameter values that most closely correspond to the data (63–69% overlap) are those which result in an average duration of immunity of at least 60 years (the best-fit is permanent immunity). With the introduction of vaccination, the inter-epidemic period increases, but this is only significant when natural immunity is relatively long-lived. In the vaccine era, under the assumption that vaccine-induced immunity lasts as long as natural immunity (Figure 2B), the parameter values that most closely correspond to the data (76–88% overlap) are those which result in an average duration of natural immunity of between 50 and 80 years (the best-fit is 60 years). If we take the extreme position that vaccine-induced immunity is very short-lived, with an average duration of 10 years (Figure 2C), then the overlap with the data is the same or worse for all but permanent immunity. When we consider the overlap between data and model output as we systematically vary both natural and vaccine-induced immunity, we find that for natural immunity greater than 40 years (excluding permanent immunity), there is some optimum duration of vaccine-induced immunity that gives rise to a high percentage overlap with the data (see Figure S2). In general, the average of the two durations appears to be between 50 and 60 years. Aggregating the results on inter-epidemic periods from both eras, we find that average durations of natural immunity of 60–100 years are consistent with the data. In addition, vaccine-induced immunity is likely to be shorter, in some cases much shorter, than natural immunity.

**Figure 3:**
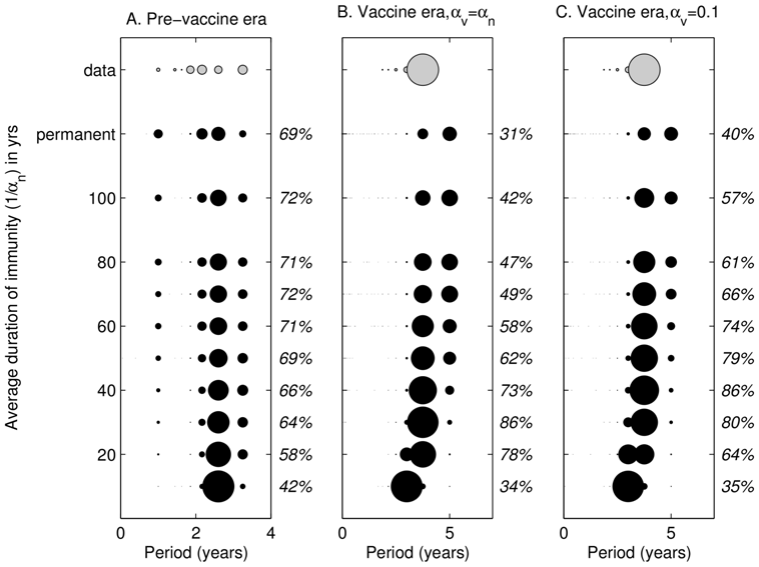
Immune-boosting model: analyses of the dominant periods of the England and Wales pertussis data (gray markers) compared to the dominant periods of stochastic realizations of the pertussis reinfection model with immune boosting (black markers), as the duration of immunity is varied. Panel A illustrates results for the pre-vaccine era, panel B for the vaccine era assuming that 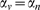, and panel C for the vaccine era fixing the average duration of vaccine-induced immunity at 10 years (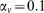). The diameter of the marker reflects the proportion of the 50 largest cities (data) or 1000 simulations (model: 50 realizations are generated for 20 different population sizes) for which spectral analysis of weekly case reports reveals that period to be the dominant signal. Any dominant period not significant at the 95% level is denoted as having a period of 0 years. The length of time series analyzed is 13 years in the pre-vaccine era, and 15 years in the vaccine era. The percentages displayed to the right of each panel are the overlap between the data and the model output. Parameter values for the model are given in Table 1, with 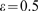. The population size, *N*, is varied from 75,000 to 1.5 million. To allow for the reintroduction of infection following extinction in a single population, we include a background force of infection of 50/million/yr (results are similar if we assume 10/million/yr). The axis representing the average duration of immunity is not to scale between 100 years (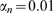) and permanent immunity (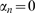).

In Figure 3, we present parallel results of the periodicity analysis for our immune-boosting model. Epidemic dynamics are most parsimonious with pre-vaccination England and Wales data when 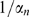 is at least 50 years (Figure 3A). In the vaccine era, if the average duration of vaccine-induced immunity is the same as that derived from natural infection, the parameter values that most closely correspond to the data (73–86% overlap) result in an average duration of natural immunity of between 20 and 40 years, as shown in Figure 3B. This range shifts to between 30 and 60 years if the average duration of vaccine-induced immunity is fixed at 10 years (Figure 3C). In general, including immune-boosting is dynamically similar to increasing the average duration of immunity because individuals experiencing immune-boosting are not infectious and contributing to transmission. However, because the effect of boosting is dependent on the repeat force of infection (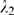), its impact varies with the duration of natural immunity and the level of vaccination.

### Critical community size

**Figure 4:**
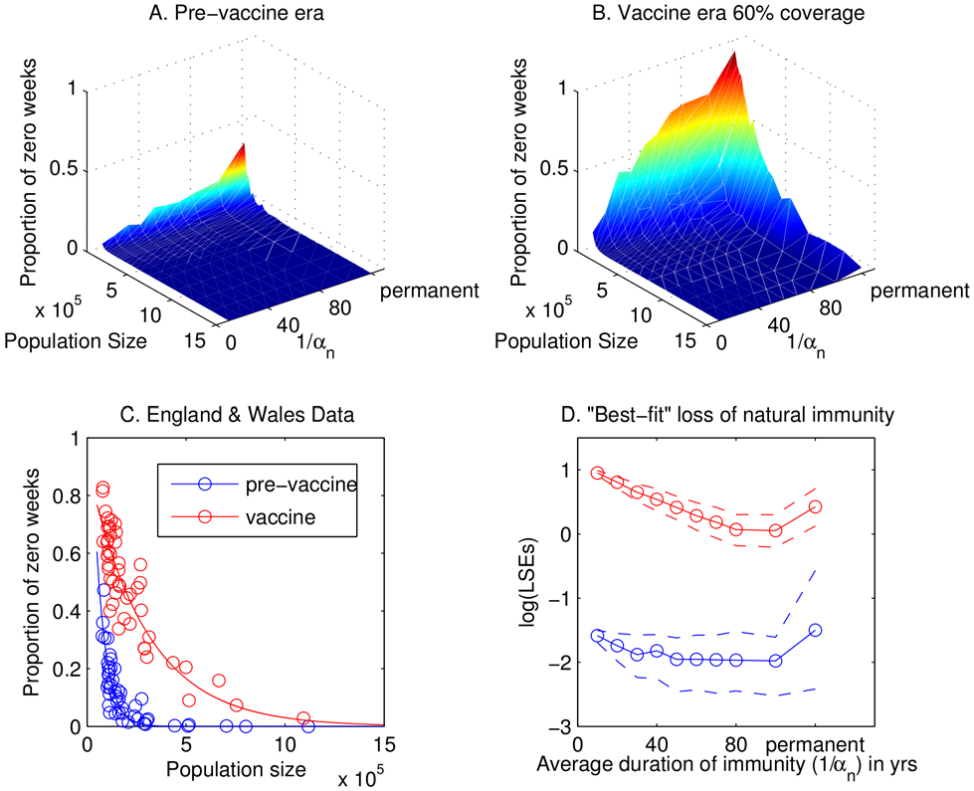
Basic model: the effects of waning immunity on critical community size. Panels A and B illustrate analyses of weekly fade-outs in the stochastic model in the pre-vaccine and vaccine era as the average duration of immunity (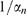) is varied. Panel C shows fade-out analyses for the England and Wales data in the pre-vaccine (blue) and vaccine (red) eras: open circles denote data points and solid lines the best-fit exponential curve. Panel D demonstrates the results of fitting model output to the fade-out curves shown in C, as assessed by the square of the residuals: the blue lines represent the pre-vaccine era; the red lines represent the vaccine era assuming that vaccine-induced immunity is lost at the rate 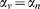. Solid lines denote averages and dashed lines indicate the 90% confidence envelope. For the stochastic model, weekly fade-outs are calculated as the average number of weeks per year with zero case reports, assuming a 15% primary reporting rate and 1% secondary reporting rate, averaged over 50 realizations for each population size. Parameter values for the model are given in Table 1, with 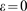. In panels A, B and D, the axis representing the average duration of immunity is not to scale between 100 years (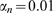) and permanent immunity (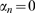).

Next we explore whether critical community size (CCS) can provide us with a further signature of waning immunity which may be detected in the data. For both data and model output, we plot a measure of the extinction frequency of the disease (fade-outs) against population size. We considered two different definitions and both produced similar results: the one we present here is the proportion of weeks with zero cases; the other measure we considered is the number of times at least 3 consecutive weeks have zero cases per epidemic. In the pre-vaccine era, analyses of fade-outs in the stochastic basic model demonstrate that the CCS increases gradually as the duration of naturally acquired immunity increases (Figure 4A). Analyses of the England and Wales data suggest a CCS of between 150,000 and 250,000 (blue markers, Figure 4C). The extremes of very rapid loss of immunity or permanent immunity result in CCSs slightly below or above this range, respectively. When vaccination is implemented, the CCS increases for all durations of immunity, except that the increase is more dramatic as the duration of immunity increases (Figure 4B). In the vaccine era, data from England and Wales suggest a CCS of between 800,000 and 1 million (red markers, Figure 4C), which is consistent with a substantial period of immunity but not permanent immunity.

Given the immunity parameters that we used in our investigations, we also determine which parameter generates a distribution of fade-outs that most closely resembles the data. We quantify this by fitting an exponential curve to the data in the two eras and then asking how well this curve fits the fade-outs predicted by the stochastic model, as assessed by the square of the residuals (see Figure 4D). We find the results to be inconclusive in the pre-vaccine era (blue lines) because there is not enough of a distinction between the fade-out profiles. In the vaccine-era (assuming 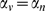), the results are quite different: the average duration of immunity that leads to the smallest error is 80 or 100 years. This error is significantly smaller than that for all durations of 40 years and below. Similar conclusions are reached if we fix 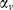 at the best-fit value from the periodicity data. This result is also robust to variations in the reporting rate of repeat infections. In fact, the lowest errors are obtained by discounting all case reports of repeat infections.

**Figure 5:**
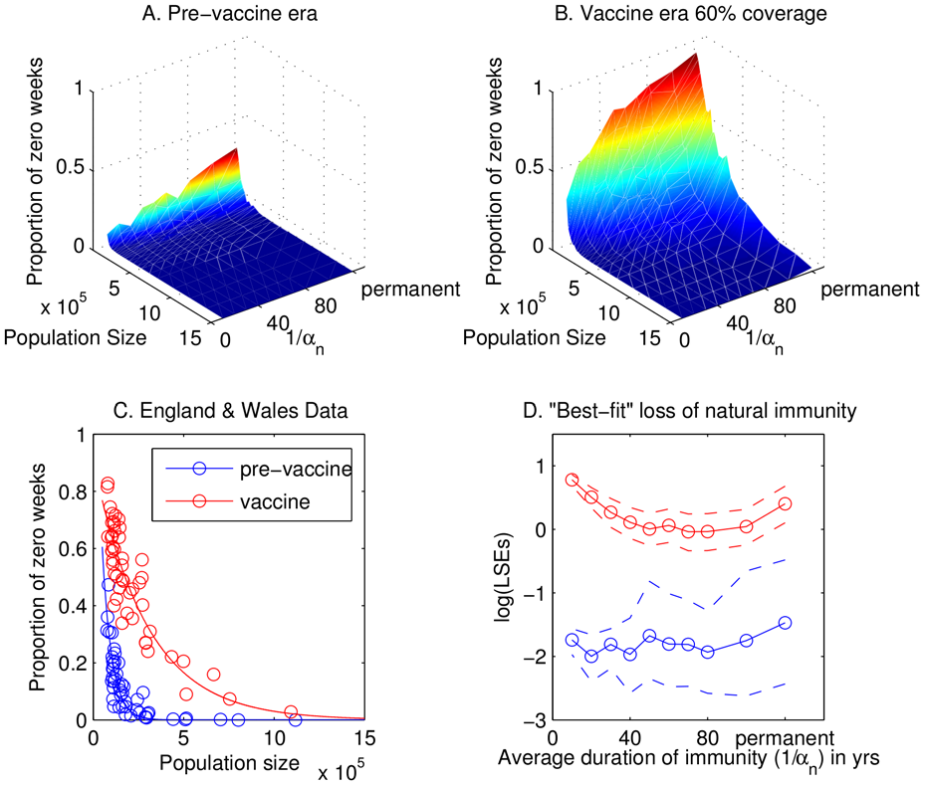
Immune-boosting model: the effects of waning immunity on critical community size. Panels A and B illustrate analyses of weekly fade-outs in the stochastic model in the pre-vaccine and vaccine era as the average duration of immunity (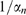) is varied. Panel C shows fade-out analyses for the England and Wales data in the pre-vaccine (blue) and vaccine (red) eras: open circles denote data points and solid lines the best-fit exponential curve. Panel D demonstrates the results of fitting model output to the fade-out curves shown in C, as assessed by the square of the residuals: the blue lines represent the pre-vaccine era; the red lines represent the vaccine era assuming that vaccine-induced immunity is lost at the rate 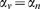. Solid lines denote averages and dashed lines indicate the 90% confidence envelope. For the stochastic model, weekly fade-outs are calculated as the average number of weeks per year with zero case reports, assuming a 15% primary reporting rate and 1% secondary reporting rate, averaged over 50 realizations for each population size. Parameter values for the model are given in Table 1, with 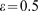. In panels A, B and D, the axis representing the average duration of immunity is not to scale between 100 years (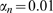) and permanent immunity (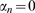).

The results of our analyses of the model with immune boosting (Figure 5) are qualitatively very similar to those for the basic model (Figure 4). The most notable difference is observed in the vaccine era, where we obtain the closest agreement with the England & Wales data (resulting in the lowest squared residuals) for a much wider range of durations of immunity: between 40 and 100 years.

### Robustness to changes in transmission parameters

For the basic model, together with the specific transmission parameters investigated above, it appears that natural immunity of an average duration of between 60 and 100 years gives the most parsimonious fit with the data as measured by inter-epidemic period and fade-out profile. We are also interested in understanding how model predictions change as we alter key transmission parameters that are difficult to estimate empirically. The parameter 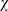 represents the ratio of the contact rate between children and adults to the average contact rate between children. Decreasing the parameter to 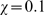 results in the same estimate of 60–100 years for the duration of natural immunity (Figures S3 and S4). The parameter 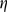 represents the relative infectiousness of repeat infections to primary infections; decreasing 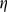 reduces the influence of repeat infections on the transmission process by reducing their contribution to the force of infection, which is dynamically very similar to increasing the immune-boosting parameter 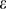. If 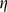 is close to zero, then primary infections are responsible for almost all transmission so the duration of immunity only plays a role in the prevalence of repeat infections. Because asymptomatic infections are almost certain to go undetected, it is unlikely that data can distinguish between different durations of immunity in this case. However, if repeat infections are such that 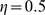, the same analyses carried out for 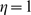 give similar results (Figures S5 and S6), with the most parsimonious range of duration of natural immunity shifting to 30–80 years. As is the case for the immune-boosting model when 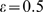.

## Discussion

Obtaining an accurate assessment of the duration of pertussis immunity is essential for informing pertussis vaccination policy [35]. In this paper, we have attempted to explore this question by interrogating transmission models to ascertain the duration of immunity that is most parsimonious with historical case notification data from England & Wales. We find that, irrespective of model choice, assuming a very short duration of natural immunity (on average less than 30 years) or permanent immunity generates predictions inconsistent with the pre-vaccine and vaccine era data from England and Wales. Shorter durations of immunity to pertussis lead to no increase in the inter-epidemic period and only a small increase in the CCS. Permanent immunity to pertussis results in a dramatic increase in inter-epidemic period and CCS. Our analyses found that a range of durations of naturally acquired immunity is consistent with the pre-vaccine and vaccine era data. If repeat infections are as infectious as primary infections with no immune-boosting then this range is 60–100 years, if they are half as infectious or 50% lead to immune-boosting infections, then this range is 30–80 years. These values are robust to changes in primary-repeat contact rates and variation in the reporting rate of repeat infections.

**Figure 6:**
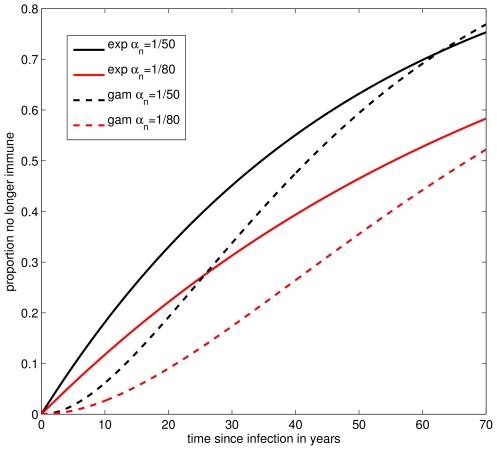
The proportion of individuals who have lost immunity as a function of time since infection, under different assumptions about the average duration of naturally acquired immunity (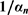) and the distribution of the immune period. The gamma-distributed periods are both with shape parameter *k* = 2, equivalent to assuming two sub-classes.

Our estimates of the average duration of natural immunity are somewhat higher than those reported in the epidemiological literature [21]. This may be in part because it is difficult to conduct a study to detect and fully sample the entire distribution of waning immunity periods amongst individuals in a population. Second, we have made a key assumption about waning immunity in our model that our predictions may rely on. The assumption, which is inherent to the standard SEIRS models, is that duration of immunity is exponentially distributed. Therefore, there is substantial variance around the mean (the coefficient of variation is 1) and many individuals will lose immunity quickly and some never at all. If we consider the time taken for 25% of the population to lose immunity, estimates of the average duration of immunity between 50 and 80 years would predict that this lies in the range 14–23 years (see Figure 6). Moreover, more than 10% of the population would have lost immunity within 10 years, which is not in contradiction with clinical reports.

We extended both models to consider a gamma-distributed immune period (with two classes, leading to a coefficient of variation of 0.7). This model was less parsimonious with the data, especially in the pre-vaccine era when it predicted longer inter-epidemic periods and higher CCSs (Figures S7, S8, S9 and S10). What these results suggest is that pertussis immunity is inherently variable, and efforts to understand waning immunity of pertussis require knowledge of the distribution of immune periods.

Unfortunately, our analyses are less conclusive about the average duration of vaccine-derived immunity. However, for the range of natural immunity consistent with the pre-vaccine era data, the corresponding durations of vaccine-derived immunity that give the best agreement with the data in the vaccine era are generally shorter than the duration of natural immunity (and are very short for the longest durations of natural immunity). Parsing out the effects of different durations of vaccine-derived immunity will require longer time series and potentially better data on “silent” repeat infections. This could be approached by considering longer datasets from the vaccine era. However, later perturbations in vaccine uptake (during the mid-1970s) and changes in vaccine content and protocols add further complexity to determining the duration of vaccine-induced immunity.

Our model analyses highlight a number of robust findings. Assuming that asymptomatic infections are unobserved, we find model output to be in strong agreement with empirical patterns as we (i) increase the average duration of immunity (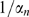), or (ii) decrease the infectiousness of repeat infections (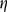) or (iii) increase the probability that, upon exposure, there is immune boosting of those whose immunity had waned (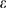). In turn, the implications of these observations are that (i) natural pertussis infection induces, on average, considerably long-lasting immunity, (ii) repeat infections contribute relatively little to the transmission cycle, and (iii) secondary exposures generate few infections (and may lead mostly to immune boosting). Taken together, these conclusions raise doubts over the impact of repeat infections in pertussis dynamics. If correct, these findings represent reasonably encouraging news for pertussis control, indicating that a reduction in prevalence (and an increase in the CCS) is possible with continued focus on increasing vaccine uptake and reducing both primary and secondary vaccine failure.

Although our study was not designed to address the issue of the recent resurgence in pertussis in certain countries, our model analyses, based on the England and Wales data, suggest that loss of natural immunity is not the primary driver. Perhaps what we should be focusing on are perturbations to pertussis dynamics in the modern era. These may include demographic changes, pathogen evolution, and perturbations in vaccine manufacture, uptake and efficiency, all of which are likely to have significant dynamical impacts. In particular, the vaccine era data considered in this study span 1958–1972 when the whole cell vaccine was in use. Extrapolation of our analyses to modern data with a variety of acellular vaccines and a booster schedule would be difficult, especially in light of known differences in the Th1/Th2 response of the whole cell and acellular vaccines [19].

**Figure 7:**
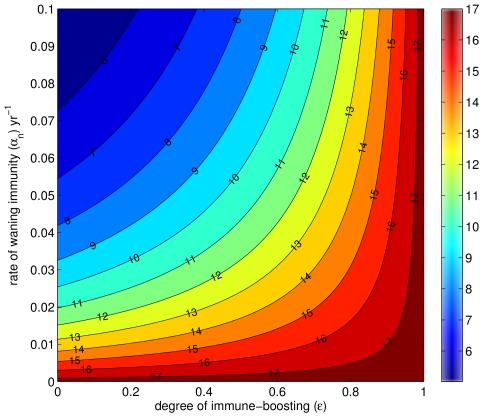
The value of *R*_0_ as the degree of immune-boosting (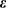) and the rate of waning immunity (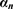) are varied for a fixed value of the mean age at primary infection. All other parameters are defined in Table 1. The mathematical formula for *R*_0_ is given in Appendix S1.

Finally, this work suggests a revision of estimates of the basic reproductive ratio, or *R*_0_, of pertussis. As mentioned above, the classic work of Anderson & May [31] has been pivotal in suggesting that the *R*_0_ of pertussis is in the range of 14–17, with the attendant control implications that vaccine coverage must be very high–in excess of approximately 93%–to achieve eradication. Their estimates were based on the well-known relationship between the mean age at (primary) infection and life expectancy, derived from models assuming permanent immunity [29],[31]. The estimates of *R*_0_ in systems where immunity is not permanent are substantially impacted by the duration of immunity [29]. Following our analyses, we find that, in the pre-vaccine era, the corresponding values of *R*_0_ fall in the range 11–15 (Figure 7). These values are lower than those commonly cited in the epidemiological literature, and paint a somewhat rosier picture regarding pertussis control. This conclusion, however, needs to be tempered by the recognition that control of infectious diseases is made much harder when using imperfect vaccines [36]–[38], especially in the face of waning immunity [39].

## Supporting Information

Figure S1 Reproduction of Figure 2 in the main text, with marker color representing the average normalized power corresponding to each dominant period. Panel A illustrates results for the pre-vaccine era, panel B for the vaccine era assuming that *α_v_* = *α_n_*, and panel C for the vaccine era fixing the average duration of vaccine-induced immunity at 10 years (*α_v_* = 0.1).
(0.36 MB TIF)

Figure S2 Basic model: the percentage overlap between the dominant periods detected in the data and those detected in the model output as both the duration of natural immunity (1/*α_n_*) and vaccine-induced immunity (1/*α_v_*) are varied in the vaccine era. Values of 1/*α_v_* above 50 years give very similar results to 1/*α_v_* = 50 (because we are only considering 15 years of time series in the vaccine era.)
(0.12 MB TIF)

Figure S3 Basic model: the effects of waning immunity on inter-epidemic period when the relative contact rate between children and adults is very low (*χ* = 0.1). Panel A illustrates results for the pre-vaccine era, panel B for the vaccine era assuming that *α_v_* = *α_n_*, and panel C for the vaccine era fixing the average duration of vaccine-induced immunity at 10 years (*α_v_* = 0.1). Compare to Figure 2 in the main text.
(0.29 MB TIF)

Figure S4 Basic model: the effects of waning immunity on critical community size when the relative contact rate between children and adults is very low (*χ* = 0.1). Panels A and B illustrate analyses of weekly fade-outs in the stochastic model in the pre-vaccine and vaccine era as the average duration of immunity (1/*α_n_*) is varied. Panel C shows fade-out analyses for the England and Wales data in the pre-vaccine (blue) and vaccine (red) eras: open circles denote data points and solid lines the best-fit exponential curve. Panel D demonstrates the results of fitting model output to the fade-out curves shown in C, as assessed by the square of the residuals: the blue lines represent the pre-vaccine era; the red lines represent the vaccine era assuming that vaccine-induced immunity is lost at the rate *α_v_* = *α_n_*. Solid lines denote averages and dashed lines indicate the 90% confidence envelope. Compare to Figure 4 in the main text.
(0.58 MB TIF)

Figure S5 Basic model: the effects of waning immunity on inter-epidemic period when repeat infections are half as infectious as primary infections (*η* = 0.5). Panel A illustrates results for the pre-vaccine era, panel B for the vaccine era assuming that *α_v_* = *α_n_*, and panel C for the vaccine era fixing the average duration of vaccine-induced immunity at 10 years (*α_v_* = 0.1). Compare to Figure 2 in the main text.
(0.29 MB TIF)

Figure S6 Basic model: the effects of waning immunity on critical community size when repeat infections are half as infectious as primary infections (*η* = 0.5). Panels A and B illustrate analyses of weekly fade-outs in the stochastic model in the pre-vaccine and vaccine era as the average duration of immunity (1/*α_n_*) is varied. Panel C shows fade-out analyses for the England and Wales data in the pre-vaccine (blue) and vaccine (red) eras: open circles denote data points and solid lines the best-fit exponential curve. Panel D demonstrates the results of fitting model output to the fade-out curves shown in C, as assessed by the square of the residuals: the blue lines represent the pre-vaccine era; the red lines represent the vaccine era assuming that vaccine-induced immunity is lost at the rate *α_v_* = *α_n_*. Solid lines denote averages and dashed lines indicate the 90% confidence envelope. Compare to Figure 4 in the main text.
(0.61 MB TIF)

Figure S7 Basic model: the effects of waning immunity on inter-epidemic period when the immune class *R* is gamma-distributed with *k* = 2. Panel A illustrates results for the pre-vaccine era, panel B for the vaccine era assuming that *α_v_* = *α_n_*, and panel C for the vaccine era fixing the average duration of vaccine-induced immunity at 10 years (*α_v_* = 0.1). Compare to Figure 2 in the main text.
(0.29 MB TIF)

Figure S8 Basic model: the effects of waning immunity on critical community size when the immune class *R* is gamma-distributed with *k* = 2. Panels A and B illustrate analyses of weekly fade-outs in the stochastic model in the pre-vaccine and vaccine era as the average duration of immunity (1/*α_n_*) is varied. Panel C shows fade-out analyses for the England and Wales data in the pre-vaccine (blue) and vaccine (red) eras: open circles denote data points and solid lines the best-fit exponential curve. Panel D demonstrates the results of fitting model output to the fade-out curves shown in C, as assessed by the square of the residuals: the blue lines represent the pre-vaccine era; the red lines represent the vaccine era assuming that vaccine-induced immunity is lost at the rate *α_v_* = *α_n_*. Solid lines denote averages and dashed lines indicate the 90% confidence envelope. Compare to Figure 4 in the main text.
(0.69 MB TIF)

Figure S9 Immune-boosting model: the effects of waning immunity on inter-epidemic period when the immune class *R* is gamma-distributed with *k* = 2. Panel A illustrates results for the pre-vaccine era, panel B for the vaccine era assuming that *α_v_* = *α_n_*, and panel C for the vaccine era fixing the average duration of vaccine-induced immunity at 10 years (*α_v_* = 0.1). Compare to Figure 3 in the main text.
(0.29 MB TIF)

Figure S10 Immune-boosting model: the effects of waning immunity on critical community size when the immune class *R* is gamma-distributed with *k* = 2. Panels A and B illustrate analyses of weekly fade-outs in the stochastic model in the pre-vaccine and vaccine era as the average duration of immunity (1/*α_n_*) is varied. Panel C shows fade-out analyses for the England and Wales data in the pre-vaccine (blue) and vaccine (red) eras: open circles denote data points and solid lines the best-fit exponential curve. Panel D demonstrates the results of fitting model output to the fade-out curves shown in C, as assessed by the square of the residuals: the blue lines represent the pre-vaccine era; the red lines represent the vaccine era assuming that vaccine-induced immunity is lost at the rate *α_v_* = *α_n_*. Solid lines denote averages and dashed lines indicate the 90% confidence envelope. Compare to Figure 5 in the main text.
(0.69 MB TIF)

Figure S11 Basic model: qualitatively similar findings when the number of stochastic realizations is increased to 500 replicates per population size. Panel A illustrates periodicity results for the pre-vaccine era, panel B for the vaccine era assuming that *α_v_* = *α_n_*, and panel C for the vaccine era fixing the average duration of vaccine-induced immunity at 10 years (*α_v_* = 0.1). Panels D and E illustrate analyses of weekly fade-outs in the stochastic model in the pre-vaccine and vaccine era as the average duration of immunity (1/*α_n_*) is varied. Panel F shows fade-out analyses for the England and Wales data in the pre-vaccine (blue) and vaccine (red) eras: open circles denote data points and solid lines the best-fit exponential curve. Panel G demonstrates the results of fitting model output to the fade-out curves shown in F, as assessed by the square of the residuals: the blue lines represent the pre-vaccine era; the red lines represent the vaccine era assuming that vaccine-induced immunity is lost at the rate *α_v_* = *α_n_*. Solid lines denote averages and dashed lines indicate the 90% confidence envelope. Compare to Figures 2 and 4 in the main text.
(0.65 MB TIF)

Appendix S1 (0.03 MB PDF)
