# Supplementary figures and images for: Estimating the Duration of Pertussis Immunity Using Epidemiological Signatures
Source: PLoS Pathog. 2009 Oct 30;5(10):e1000647. doi: 10.1371/journal.ppat.1000647 (PMC2763266; doi:10.1371/journal.ppat.1000647)

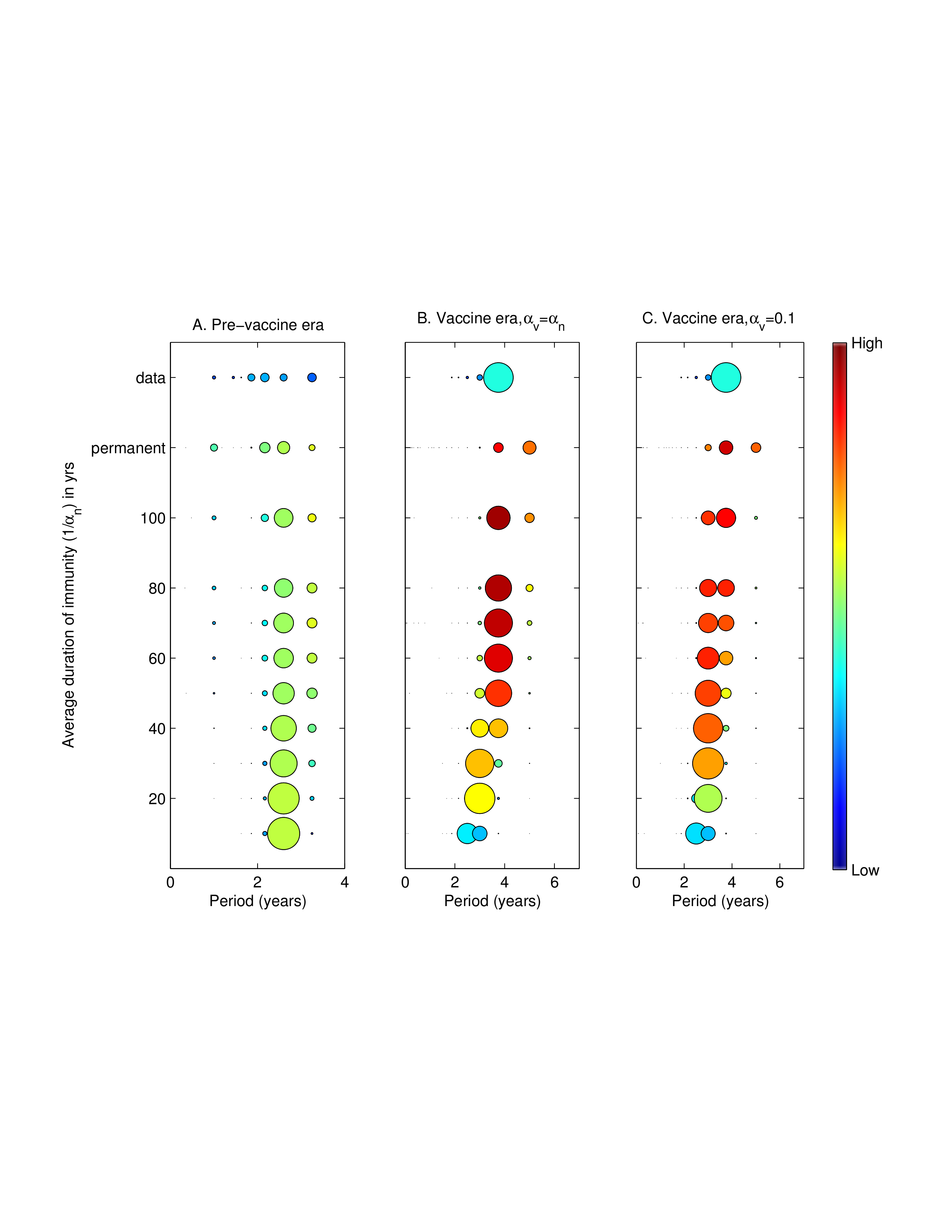

Supplement: Figure S1 — Reproduction of Figure 2 in the main text, with marker color representing the average normalized power corresponding to each dominant period. Panel A illustrates results for the pre-vaccine era, panel B for the vaccine era assuming that αv = αn, and panel C for the vaccine era fixing the average duration of vaccine-induced immunity at 10 years (αv = 0.1). (0.36 MB TIF) [file ppat.1000647.s001.tif]

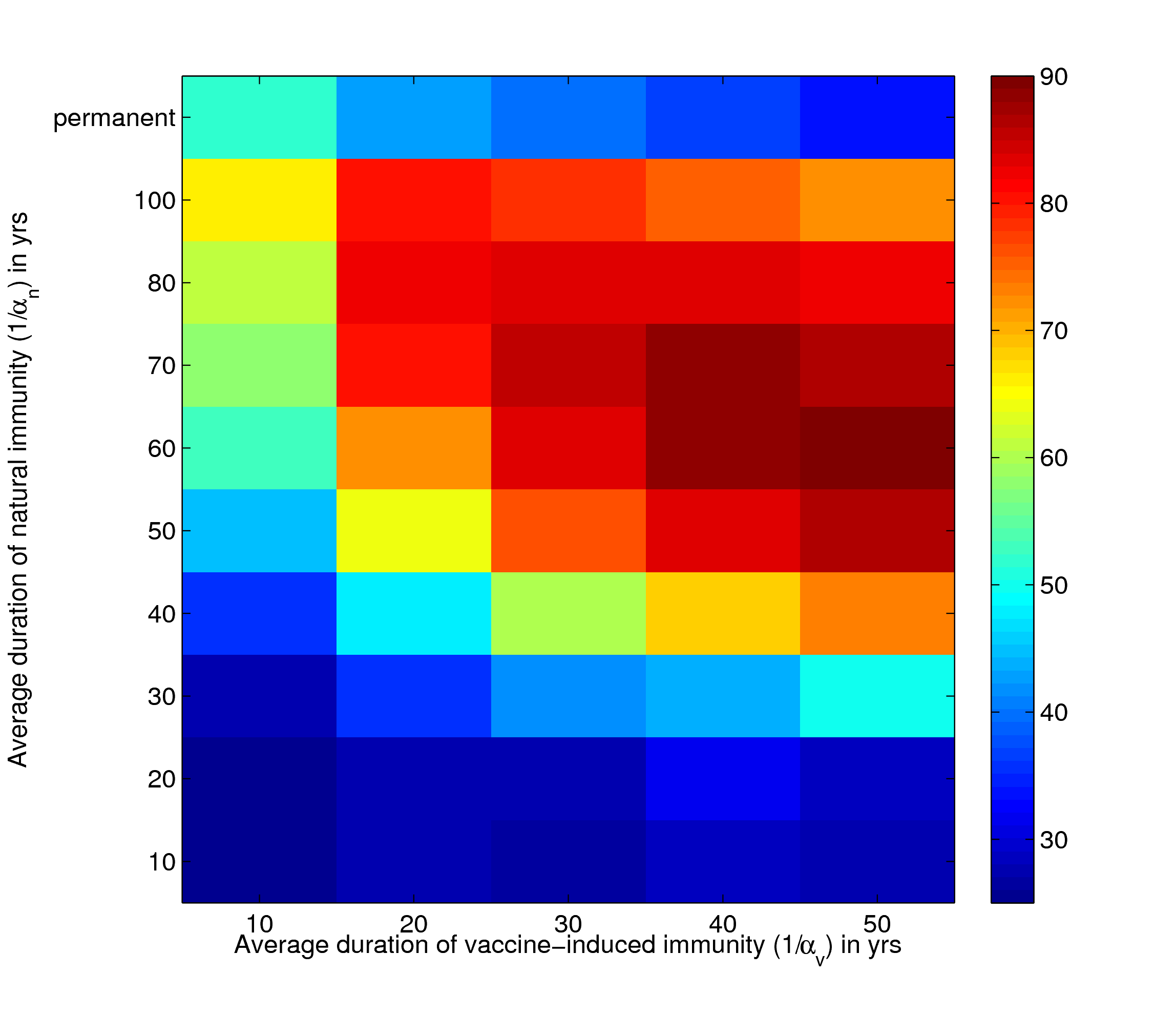

Supplement: Figure S2 — Basic model: the percentage overlap between the dominant periods detected in the data and those detected in the model output as both the duration of natural immunity (1/αn) and vaccine-induced immunity (1/αv) are varied in the vaccine era. Values of 1/αv above 50 years give very similar results to 1/αv = 50 (because we are only considering 15 years of time series in the vaccine era.) (0.12 MB TIF) [file ppat.1000647.s002.tif]

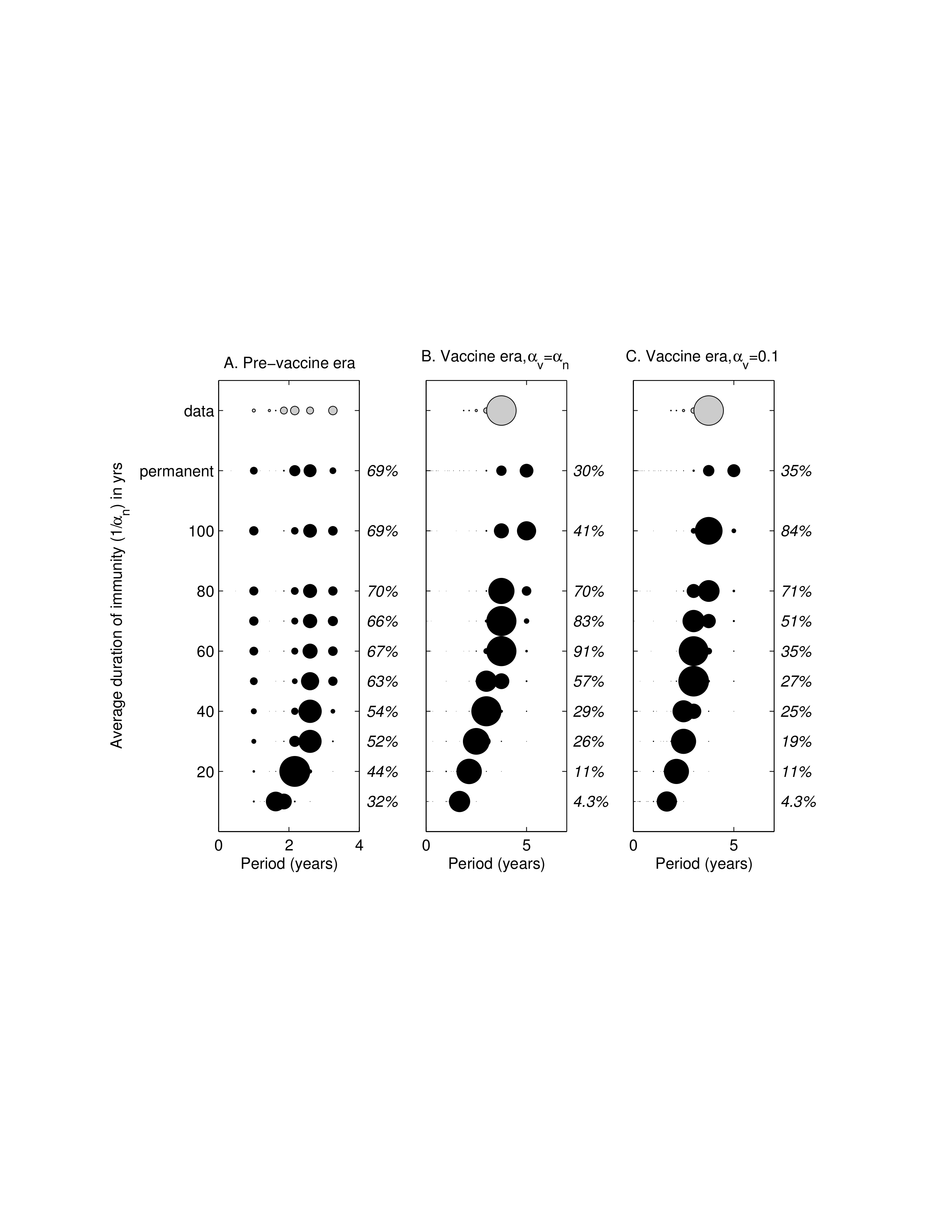

Supplement: Figure S3 — Basic model: the effects of waning immunity on inter-epidemic period when the relative contact rate between children and adults is very low (χ = 0.1). Panel A illustrates results for the pre-vaccine era, panel B for the vaccine era assuming that αv = αn, and panel C for the vaccine era fixing the average duration of vaccine-induced immunity at 10 years (αv = 0.1). Compare to Figure 2 in the main text. (0.29 MB TIF) [file ppat.1000647.s003.tif]

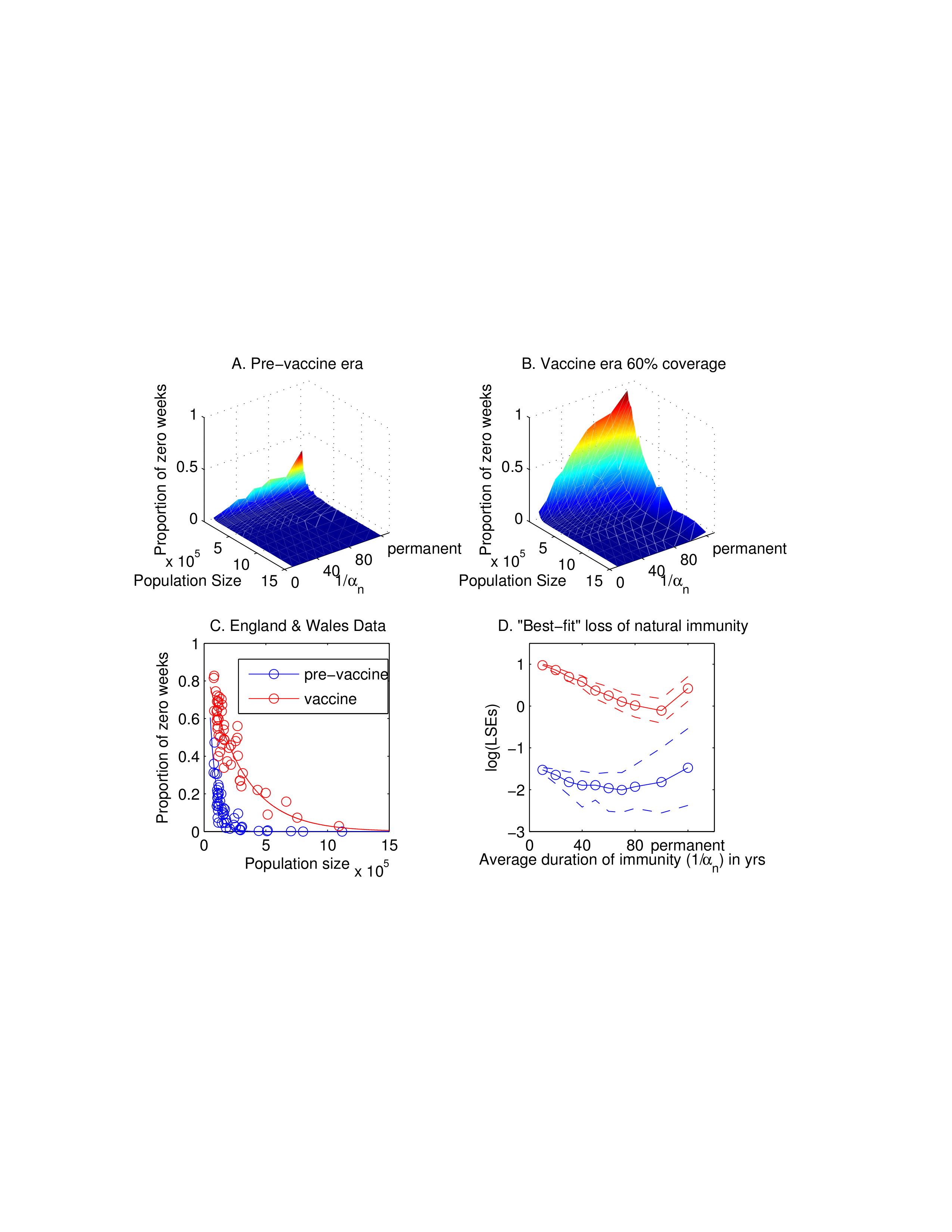

Supplement: Figure S4 — Basic model: the effects of waning immunity on critical community size when the relative contact rate between children and adults is very low (χ = 0.1). Panels A and B illustrate analyses of weekly fade-outs in the stochastic model in the pre-vaccine and vaccine era as the average duration of immunity (1/αn) is varied. Panel C shows fade-out analyses for the England and Wales data in the pre-vaccine (blue) and vaccine (red) eras: open circles denote data points and solid lines the best-fit exponential curve. Panel D demonstrates the results of fitting model output to the fade-out curves shown in C, as assessed by the square of the residuals: the blue lines represent the pre-vaccine era; the red lines represent the vaccine era assuming that vaccine-induced immunity is lost at the rate αv = αn. Solid lines denote averages and dashed lines indicate the 90% confidence envelope. Compare to Figure 4 in the main text. (0.58 MB TIF) [file ppat.1000647.s004.tif]

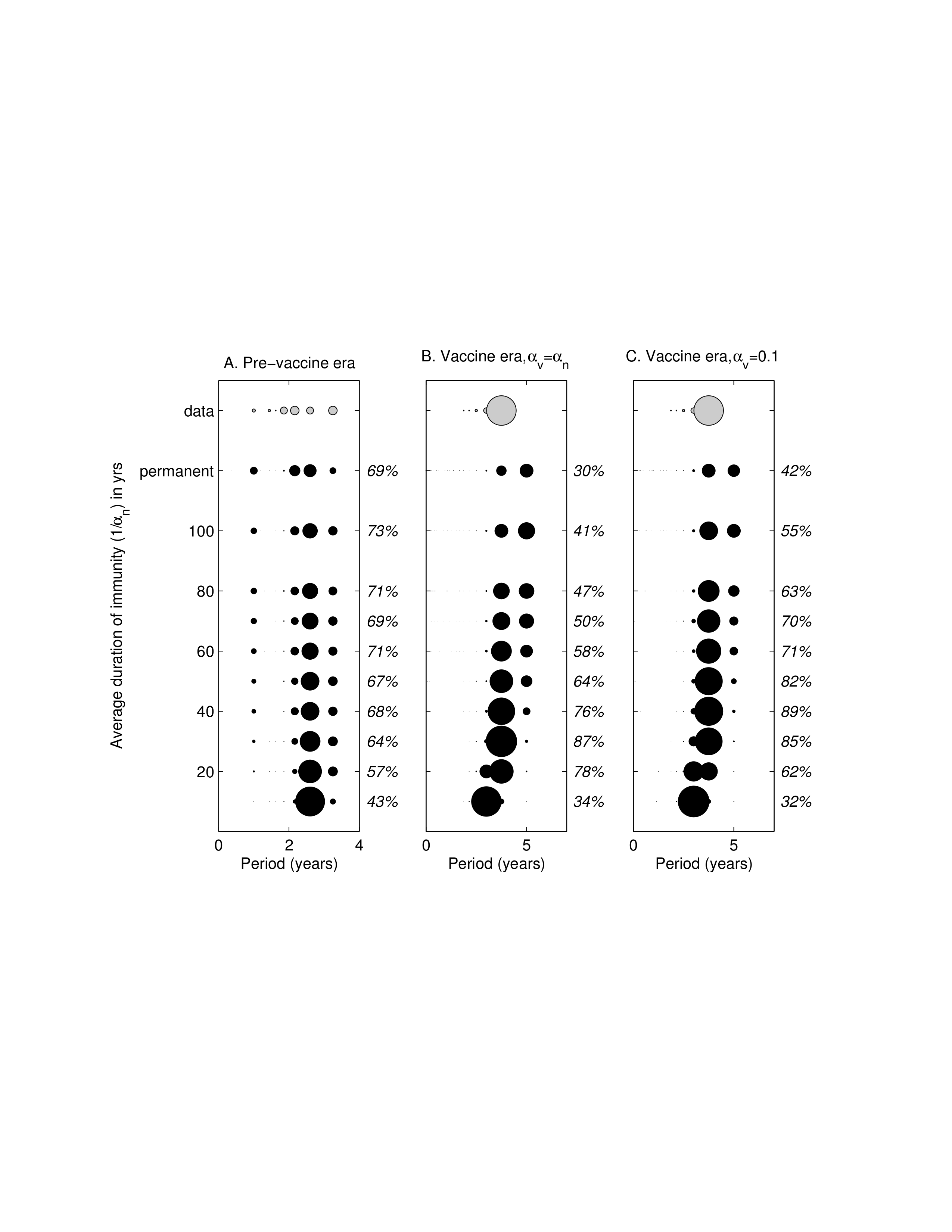

Supplement: Figure S5 — Basic model: the effects of waning immunity on inter-epidemic period when repeat infections are half as infectious as primary infections (η = 0.5). Panel A illustrates results for the pre-vaccine era, panel B for the vaccine era assuming that αv = αn, and panel C for the vaccine era fixing the average duration of vaccine-induced immunity at 10 years (αv = 0.1). Compare to Figure 2 in the main text. (0.29 MB TIF) [file ppat.1000647.s005.tif]

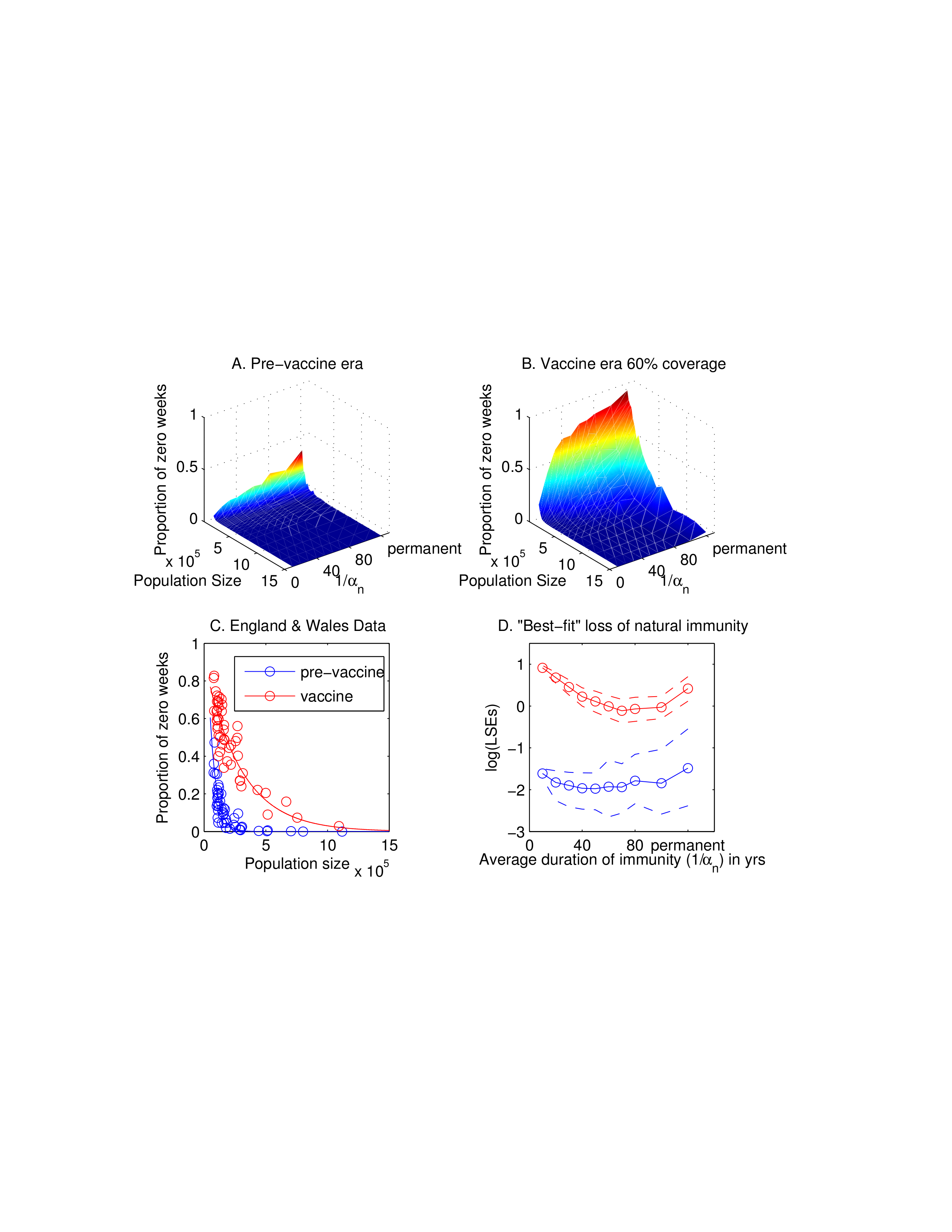

Supplement: Figure S6 — Basic model: the effects of waning immunity on critical community size when repeat infections are half as infectious as primary infections (η = 0.5). Panels A and B illustrate analyses of weekly fade-outs in the stochastic model in the pre-vaccine and vaccine era as the average duration of immunity (1/αn) is varied. Panel C shows fade-out analyses for the England and Wales data in the pre-vaccine (blue) and vaccine (red) eras: open circles denote data points and solid lines the best-fit exponential curve. Panel D demonstrates the results of fitting model output to the fade-out curves shown in C, as assessed by the square of the residuals: the blue lines represent the pre-vaccine era; the red lines represent the vaccine era assuming that vaccine-induced immunity is lost at the rate αv = αn. Solid lines denote averages and dashed lines indicate the 90% confidence envelope. Compare to Figure 4 in the main text. (0.61 MB TIF) [file ppat.1000647.s006.tif]

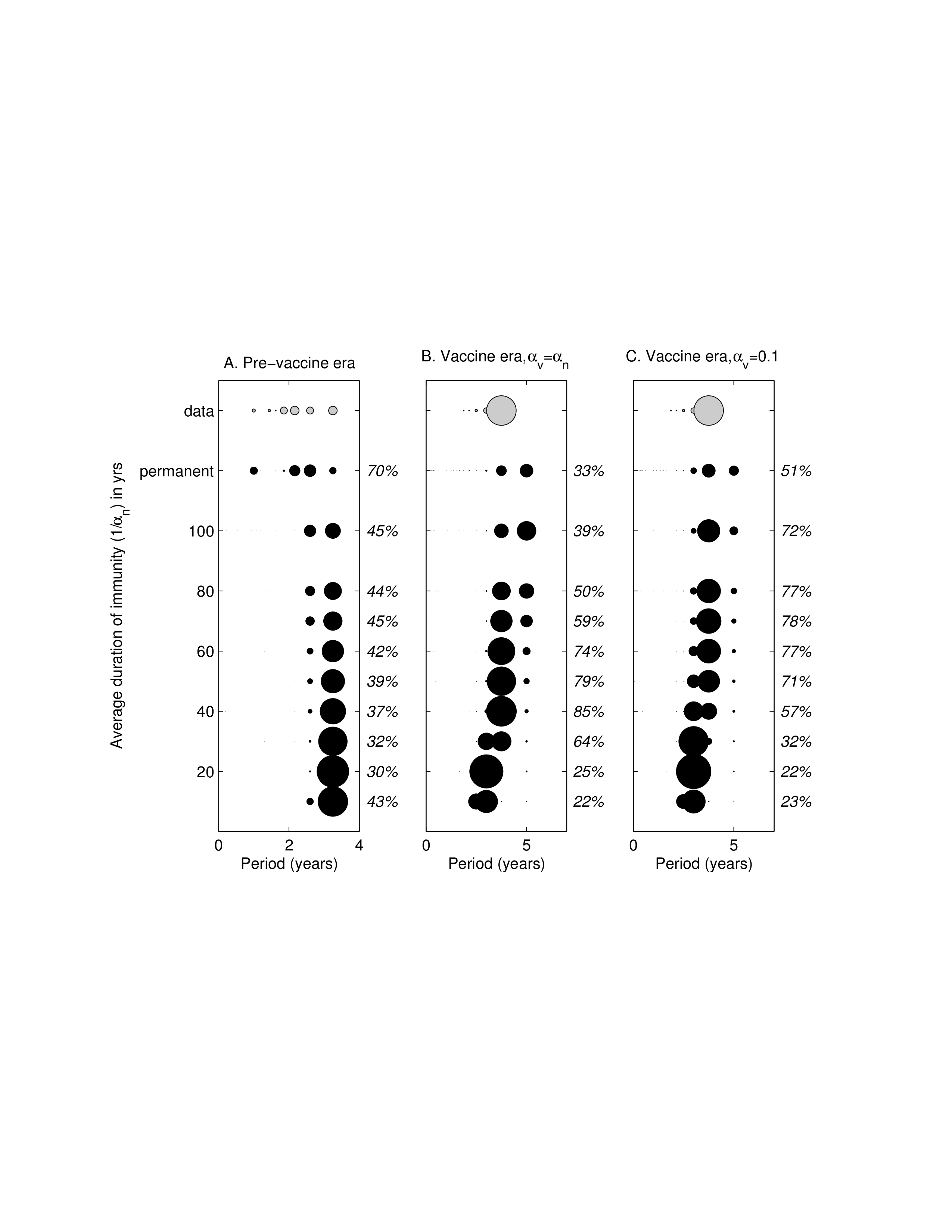

Supplement: Figure S7 — Basic model: the effects of waning immunity on inter-epidemic period when the immune class R is gamma-distributed with k = 2. Panel A illustrates results for the pre-vaccine era, panel B for the vaccine era assuming that αv = αn, and panel C for the vaccine era fixing the average duration of vaccine-induced immunity at 10 years (αv = 0.1). Compare to Figure 2 in the main text. (0.29 MB TIF) [file ppat.1000647.s007.tif]

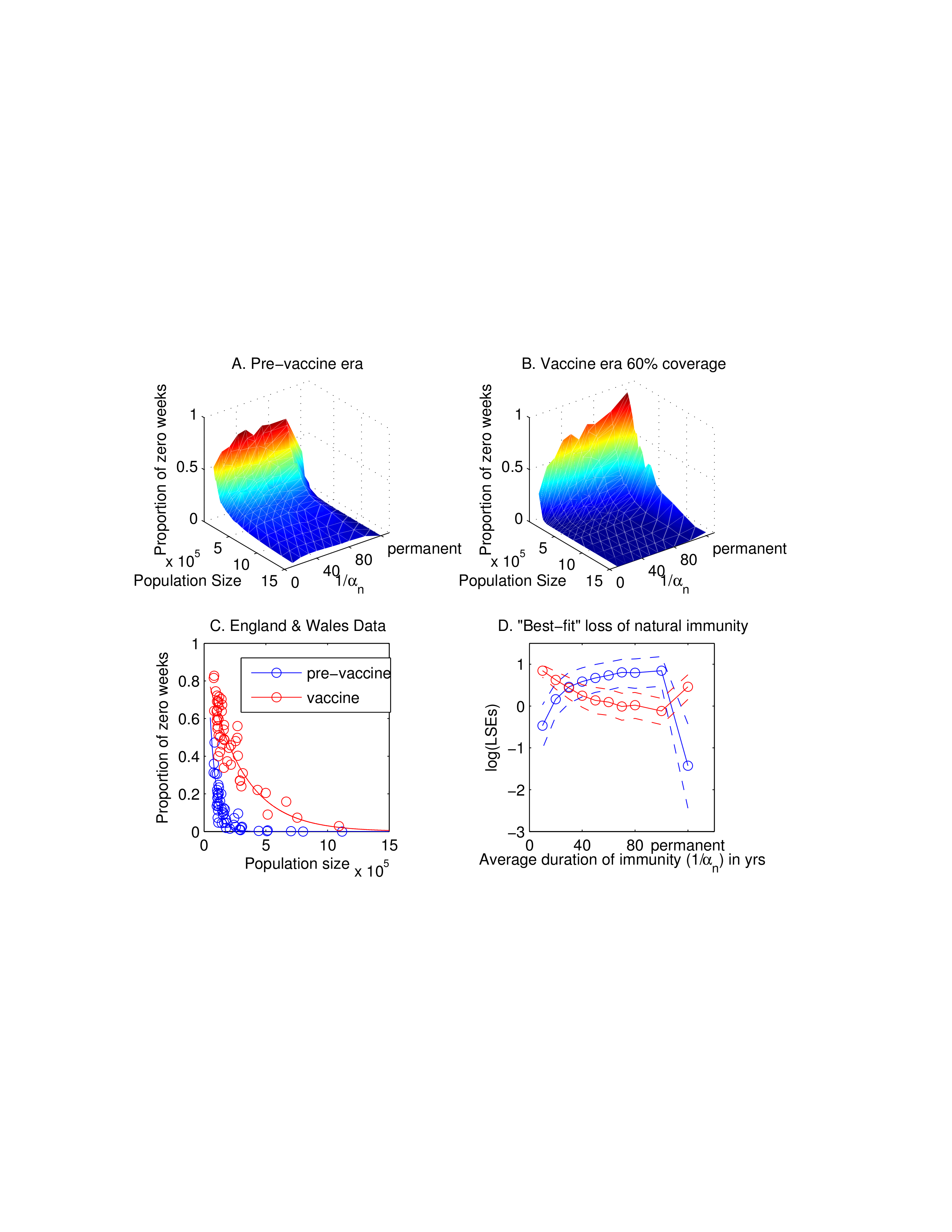

Supplement: Figure S8 — Basic model: the effects of waning immunity on critical community size when the immune class R is gamma-distributed with k = 2. Panels A and B illustrate analyses of weekly fade-outs in the stochastic model in the pre-vaccine and vaccine era as the average duration of immunity (1/αn) is varied. Panel C shows fade-out analyses for the England and Wales data in the pre-vaccine (blue) and vaccine (red) eras: open circles denote data points and solid lines the best-fit exponential curve. Panel D demonstrates the results of fitting model output to the fade-out curves shown in C, as assessed by the square of the residuals: the blue lines represent the pre-vaccine era; the red lines represent the vaccine era assuming that vaccine-induced immunity is lost at the rate αv = αn. Solid lines denote averages and dashed lines indicate the 90% confidence envelope. Compare to Figure 4 in the main text. (0.69 MB TIF) [file ppat.1000647.s008.tif]

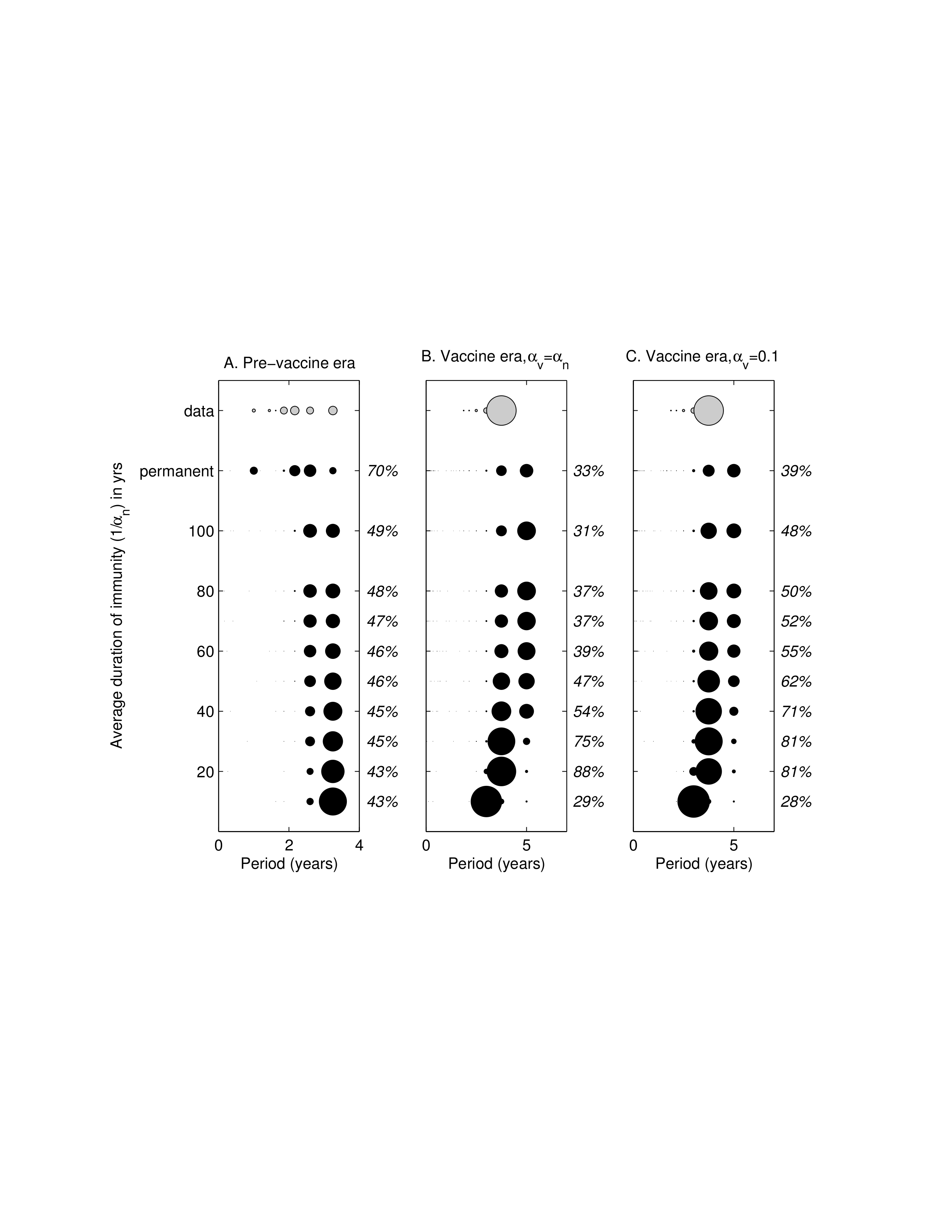

Supplement: Figure S9 — Immune-boosting model: the effects of waning immunity on inter-epidemic period when the immune class R is gamma-distributed with k = 2. Panel A illustrates results for the pre-vaccine era, panel B for the vaccine era assuming that αv = αn, and panel C for the vaccine era fixing the average duration of vaccine-induced immunity at 10 years (αv = 0.1). Compare to Figure 3 in the main text. (0.29 MB TIF) [file ppat.1000647.s009.tif]

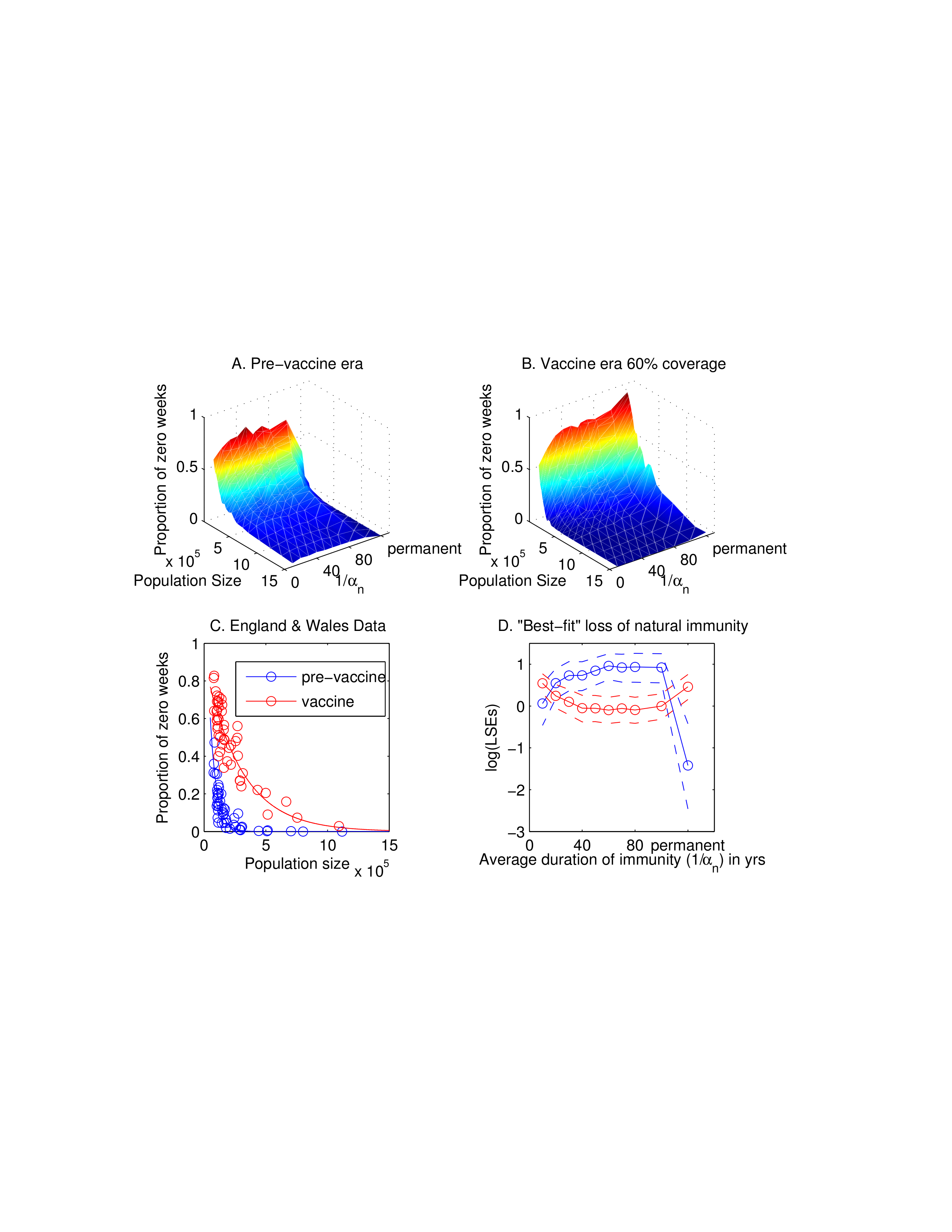

Supplement: Figure S10 — Immune-boosting model: the effects of waning immunity on critical community size when the immune class R is gamma-distributed with k = 2. Panels A and B illustrate analyses of weekly fade-outs in the stochastic model in the pre-vaccine and vaccine era as the average duration of immunity (1/αn) is varied. Panel C shows fade-out analyses for the England and Wales data in the pre-vaccine (blue) and vaccine (red) eras: open circles denote data points and solid lines the best-fit exponential curve. Panel D demonstrates the results of fitting model output to the fade-out curves shown in C, as assessed by the square of the residuals: the blue lines represent the pre-vaccine era; the red lines represent the vaccine era assuming that vaccine-induced immunity is lost at the rate αv = αn. Solid lines denote averages and dashed lines indicate the 90% confidence envelope. Compare to Figure 5 in the main text. (0.69 MB TIF) [file ppat.1000647.s010.tif]

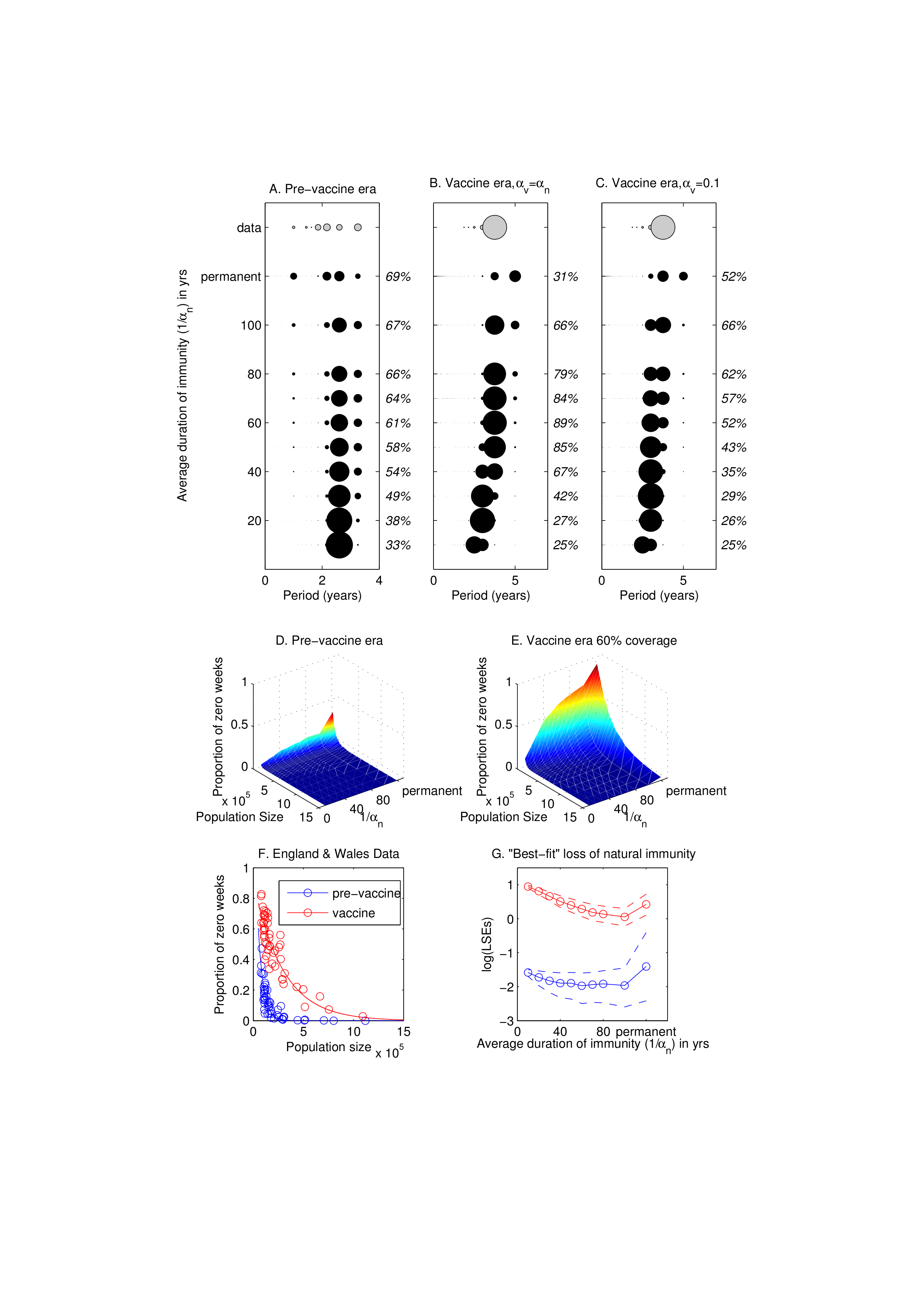

Supplement: Figure S11 — Basic model: qualitatively similar findings when the number of stochastic realizations is increased to 500 replicates per population size. Panel A illustrates periodicity results for the pre-vaccine era, panel B for the vaccine era assuming that αv = αn, and panel C for the vaccine era fixing the average duration of vaccine-induced immunity at 10 years (αv = 0.1). Panels D and E illustrate analyses of weekly fade-outs in the stochastic model in the pre-vaccine and vaccine era as the average duration of immunity (1/αn) is varied. Panel F shows fade-out analyses for the England and Wales data in the pre-vaccine (blue) and vaccine (red) eras: open circles denote data points and solid lines the best-fit exponential curve. Panel G demonstrates the results of fitting model output to the fade-out curves shown in F, as assessed by the square of the residuals: the blue lines represent the pre-vaccine era; the red lines represent the vaccine era assuming that vaccine-induced immunity is lost at the rate αv = αn. Solid lines denote averages and dashed lines indicate the 90% confidence envelope. Compare to Figures 2 and 4 in the main text. (0.65 MB TIF) [file ppat.1000647.s011.tif]
