# Supplementary material for: Estimating the Duration of Pertussis Immunity Using Epidemiological Signatures
Source: PLoS Pathog. 2009 Oct 30;5(10):e1000647. doi: 10.1371/journal.ppat.1000647 (PMC2763266; doi:10.1371/journal.ppat.1000647)
Supplement: Appendix S1 — (0.03 MB PDF) [file ppat.1000647.s012.pdf]

Appendix

Calculating β_0 and R_0

In the absence of seasonal forcing ($a = 0$), the average age at primary infection, A_p, is given by

$$A_p = \frac{1}{\lambda_1^* + \mu} \ ,$$

where $\lambda_1^* = \beta_0(I_1^* + \eta\chi I_2^*)/N$, evaluated at equilibrium. Therefore, if we have independent estimates of A_p and μ, then we can estimate the primary force of infection from

$$\lambda_1^* = \frac{1}{A_p} - \mu \ .$$

Also, under the assumption that $\nu = \mu$ (and thus N is constant),

$$\frac{I_1^*}{N} = \frac{(1-p)\mu\sigma(1 - A_p\mu)}{(\sigma + \mu)(\gamma_1 + \mu)} \ .$$

We can then express I_2^*/N in terms of λ_1^* and I_1^*/N, eliminate the variables and solve the equations to find β_0 in terms of A_p and the other parameters. After some algebraic manipulation (and setting $p = 0$ to represent the pre-vaccine era):

$$\beta_0 = \frac{(\sigma + \mu)(\gamma_1 + \mu)}{\mu\sigma A_p} Y,$$

where Y is the smallest real positive solution to the following quadratic equation

$$a_0 Y^2 + a_1 Y + a_2 = 0$$

with coefficients:

$$\begin{aligned}
a_0 &= \left(\chi - \frac{\xi}{\chi}\right)\left[(C(1-\epsilon)\eta'\chi + (C-1)\epsilon - C)\,\frac{\alpha_n}{\alpha_n + \mu} + 1\right] \\
a_1 &= \frac{\mu}{\lambda_1^*} + C(1-\epsilon)\eta'\xi\frac{\alpha_n}{\alpha_n + \mu} + \left[((1-C)\epsilon + C)\frac{\alpha_n}{\alpha_n + \mu} - 1\right]\left(\chi - 2\frac{\xi}{\chi}\right) \\
a_2 &= -\frac{\mu}{\lambda_1^*} + \left[((1-C)\epsilon + C)\frac{\alpha_n}{\alpha_n + \mu} - 1\right]\frac{\xi}{\chi},
\end{aligned}$$

where $\eta' = \eta\gamma_1/\gamma_2$ and

$$C = \frac{\sigma\gamma_2}{(\sigma+\mu)(\gamma_2+\mu)} \approx 1$$

because σ and γ_2 are much larger than μ for an acute infection such as pertussis.

Therefore, substituting $C = 1$ into the expressions for the coefficients we obtain

$$\begin{aligned}
a_0 &\approx \left(\chi - \frac{\xi}{\chi}\right)\left[((1-\epsilon)\eta'\chi - 1)\frac{\alpha_n}{\alpha_n+\mu} + 1\right] \\
a_1 &\approx \frac{\mu}{\lambda_1^*} + (1-\epsilon)\eta'\xi\frac{\alpha_n}{\alpha_n+\mu} + \left[\frac{\alpha_n}{\alpha_n+\mu} - 1\right]\left(\chi - 2\frac{\xi}{\chi}\right) \\
a_2 &\approx -\frac{\mu}{\lambda_1^*} + \left[\frac{\alpha_n}{\alpha_n+\mu} - 1\right]\frac{\xi}{\chi}.
\end{aligned}$$

When average contact rates are the same, $\chi = \xi = 1$, Y simplifies to the following expression:

$$Y \approx \frac{1 - \frac{\alpha_n}{\alpha_n+\mu}(1 - A_p\mu)}{1 - [1 - \eta'(1-\epsilon)]\frac{\alpha_n}{\alpha_n+\mu}(1 - A_p\mu)}.$$

From eigenvalue calculations,

$$R_0 = \frac{L}{A_p}Y,$$

where L denotes average life expectancy $1/\mu$.

The calculations above are for exponentially-distributed infectious periods ($n = 1$). The same calculations for gamma-distributed infectious periods lead to similar expressions (but are more algebraically involved.)

Model with gamma-distributed immune period

$$
\begin{aligned}
\frac{dS_1}{dt} &= (1-p)\nu N - \lambda_1 S_1 - \mu S_1 \\
\frac{dV}{dt} &= p\nu N - (\alpha_v + \mu)V \\
\frac{dE_1}{dt} &= \lambda_1 S_1 - (\sigma + \mu)E_1 \\
\frac{dI_1^1}{dt} &= \sigma E_1 - (n\gamma_1 + \mu)I_1^1 \\
&\vdots \\
\frac{dI_1^n}{dt} &= n\gamma_1 I_1^{n-1} - (n\gamma_1 + \mu)I_1^n \\
\frac{dR^1}{dt} &= \epsilon\lambda_2 S_2 + n\gamma_1 I_1^n + n\gamma_2 I_2^n - (k\alpha_n + \mu)R^1 \\
&\vdots \\
\frac{dR^k}{dt} &= k\alpha_n R^{k-1} - (k\alpha_n + \mu)R^k \\
\frac{dS_2}{dt} &= \alpha_v V + k\alpha_n R^k - \lambda_2 S_2 - \mu S_2 \\
\frac{dE_2}{dt} &= (1-\epsilon)\lambda_2 S_2 - (\sigma + \mu)E_2 \\
\frac{dI_2^1}{dt} &= \sigma E_2 - (n\gamma_2 + \mu)I_2^1 \\
&\vdots \\
\frac{dI_2^n}{dt} &= n\gamma_2 I_2^{n-1} - (n\gamma_2 + \mu)I_2^n
\end{aligned}
$$

where $\lambda_1 = (\beta_{11}(t)I_1 + \beta_{12}I_2)/N$ and $\lambda_2 = (\beta_{21}I_1 + \beta_{22}I_2)/N$, with $I_1 = \Sigma_{i=1}^n I_1^i$ and $I_2 = \Sigma_{i=1}^n I_2^i$. The only extra parameter is k, which is the number of sub-classes of R (and is inversely related to the variance of the immune period).
